# *In vivo* targeting of cutaneous melanoma using an MSH-engineered human protein cage bearing fluorophore and MRI tracers

**DOI:** 10.1186/1479-5876-12-S1-P6

**Published:** 2014-05-06

**Authors:** Luca Vannucci, Elisabetta Falvo, Cristina Maria Failla, Miriam Carbo, Manuela Fornara, Rossella Canese, Serena Cecchetti, Lenka Rajsiglova, Dmitry Stakheev, Jiri Krizan, Alberto Boffi, Giulia Carpinelli, Veronica Morea, Pierpaolo Ceci

**Affiliations:** 1 Institute of Microbiology, Academy of Sciences of the Czech Republic (ASCR), v.v.i., Prague (CZ; 2 CNR – National Research Council of Italy, Institute of Molecular Biology and Pathology, Rome (Italy; 3 Molecular and Cell Biology Laboratory, IDI-IRCCS, Rome (Italy; 4 Department of Biochemical Sciences “A. Rossi Fanelli”, University of Rome “Sapienza” (Italy; 5 Department of Molecular and Cellular Imaging, Istituto Superiore di Sanità, Italy; 6 Center for Life Nano Science at Sapienza, Istituto Italiano di Tecnologia (IIT

## Background

Nanoparticle (NP)-based materials are very promising agents for enhancing cancer diagnosis and treatment. Once functionalized for selective targeting of tumor expressed molecules, they can specifically deliver drugs and diagnostic molecules inside tumor cells.

## Materials and methods

In the present work, we evaluated the in vivo melanoma-targeting ability of a nanovector (HFt-MSH-PEG) based on human protein ferritin (HFt), functionalized with both melanoma-targeting melanoma stimulating hormone (α-MSH) and stabilizing poly(ethylene glycol) (PEG) molecules. We used two independent and complementary techniques, such as whole-specimen confocal microscopy and magnetic resonance imaging, to detect the in vivo localization of NP constructs endowed with suitable tracers (i.e., fluorophores or magnetic metals).

## Results

Targeted HFt-MSH-PEG NPs were shown to accumulate persistently at the level of primary melanoma and with high selectivity with respect to other organs. Melanoma localization of untargeted HFt-PEG NPs, lacking the α-MSH moiety, was less pronounced and disappeared after a few days. Further, HFt-MSH-PEG NPs accumulated to a significantly lower extent and with a different distribution in a diverse type of tumor (TS/A adenocarcinoma), which does not express α-MSH receptors. Finally, in a spontaneous lung metastasis model, HFt-MSH-PEG NPs localized at the metastasis level as well.

## Conclusions

These results point at HFt-MSH-PEG NPs as suitable carriers for selective in vivo delivery of diagnostic or therapeutic agents to cutaneous melanoma.

